# Temporal evaluation of commitment to sexual development in *Plasmodium falciparum*

**DOI:** 10.1186/1475-2875-12-134

**Published:** 2013-04-22

**Authors:** Christopher L Peatey, Matthew WA Dixon, Donald L Gardiner, Katharine R Trenholme

**Affiliations:** 1 Malaria Biology Laboratory, Queensland Institute of Medical Research, 300 Herston Rd, Herston, QLD 4006, Australia; 2 School of Molecular and Microbial Sciences, University of Queensland, Queensland, Australia; 3 School of Biomolecular and Physical Sciences, Griffith University, Queensland, Australia; 4 School of Medicine, University of Queensland, Queensland, Australia; 5 Department of Biochemistry and Molecular Biology, Bio21 Molecular Science and Biotechnology Institute, and ARC Centre of Excellence for Coherent X-ray Science, University of Melbourne, Parkville, VIC 3010, Australia

**Keywords:** *Plasmodium falciparum*, Malaria, Gametocyte

## Abstract

**Background:**

The production of gametocytes is essential for transmission of malaria parasites from the mammalian host to the mosquito vector. However the process by which the asexual blood-stage parasite undergoes commitment to sexual development is not well understood. This process is known to be sensitive to environmental stimuli and it has been suggested that a G protein dependent system may mediate the switch, but there is little evidence that the *Plasmodium falciparum* genome encodes heterotrimeric G proteins. Previous studies have indicated that the malaria parasite can interact with endogenous erythrocyte G proteins, and other components of the cyclic nucleotide pathway have been identified in *P. falciparum*. Also, the polypeptide cholera toxin, which induces commitment to gametocytogenesis is known to catalyze the ADP-ribosylation of the α_s_ class of heterotrimeric G protein α subunits in mammalian systems has been reported to detect a number of G_α_ subunits in *P. falciparum-*infected red cells.

**Methods:**

Cholera toxin and Mas 7 (a structural analogue of Mastoparan) were used to assess the role played by putative G protein signalling in the commitment process, both are reported to interact with different components of classical Gαs and Gαi/o signalling pathways. Their ability to induce gametocyte production in the transgenic *P. falciparum* line Pfs16-GFP was determined and downstream effects on the secondary messenger cAMP measured.

**Results:**

Treatment of parasite cultures with either cholera toxin or MAS 7 resulted in increased gametocyte production, but only treatment with MAS 7 resulted in a significant increase in cAMP levels. This indicates that MAS 7 acts either directly or indirectly on the *P. falciparum* adenylyl cyclase.

**Conclusion:**

The observation that cholera toxin treatment did not affect cAMP levels indicates that while addition of cholera toxin does increase gametocytogenesis the method by which it induces increased commitment is not immediately obvious, except that is unlikely to be via heterotrimeric G proteins.

## Background

A switch from asexual to sexual development resulting in the production of gametocytes is essential for transmission of the malaria parasite to the mosquito vector but the mechanisms behind this switch are still poorly understood. Gametocyte production generally increases in response to conditions negatively affecting asexual multiplication reviewed in [1], and can be induced *in vitro* by various agents including erythrocyte lysate [2], spent culture media (conditioned media) [3] and 8-bromo cAMP [4], the polypeptide cholera toxin [5], and many commonly used anti-malarials, such as chloroquine and Fansidar reviewed in [6].

There is strong evidence that commitment to gametocytogenesis is sensitive to environmental conditions suggesting that a signalling pathway between the environment and the parasite must be involved. Detection of such changes might occur via classical signal transduction pathways involving growth factors or other environmental signals. Classical eukaryotic signal transduction pathways have a number of similar features, and operate through a limited number of cellular effector mechanisms [7]. The most widely used mechanism is signalling through G protein coupled receptors (GPCR). GPCR are an extensive group of hormone, pheromone, neurotransmitter, odorant and light receptors that transduce extracellular signals by interaction with heterotrimeric G proteins or through less well-defined Mitogen Activated Protein Kinase-dependent pathways [7,8]. Heterotrimeric G proteins consist of an α-subunit and a βγ − complex. Four major families of α − subunits - Gαs, Gαi/o, Gαq11 and Gα12/13 have been described in eukaryotic cells and each specifies a distinct set of downstream signals. While there is little evidence that the *Plasmodium falciparum* genome encodes heterotrimeric G proteins [9,10], previous studies have indicated that the malaria parasite can interact with endogenous erythrocyte G proteins [11].

Nonetheless, other components of the cyclic nucleotide pathway have been identified in *P. falciparum*, including adenylyl and guanylyl cyclases and their effector kinases [9,12]. The polypeptide cholera toxin (CT), which is known to catalyze the ADP-ribosylation of the α_s_ class of heterotrimeric G protein α subunits in mammalian systems, has been reported to detect a number of G_α_ subunits in *P. falciparum-*infected red cells [13] and induces commitment to gametocytogenesis [5]. However, direct evidence that CT results in increased cAMP levels or that the human homologues of heterotrimeric G proteins interact canonically with *P. falciparum* adenylyl cyclases is lacking [9].

In order to further assess the role played by putative G protein signalling in the commitment process, two different compounds reported to interact with different components of classical Gαs and Gαi/o signalling pathways were used. In mammalian systems these pathways generally act in opposition to each other with the Gαs pathway activating and the Gαi/o pathway inhibiting adenylyl cyclase signalling. In order to investigate signalling through the alternate Gαi/o pathway the ability of the peptide MAS 7, a structural analogue of Mastoparan and a known activator of heterotrimeric Gαi/o proteins and downstream effectors [14] to induce or inhibit gametocytogenesis, was determined.

To accurately determine the effect of MAS 7 and CT on commitment, an assay using a green fluorescent protein (GFP) chimera of the early gametocyte marker Pfs16 [15,16] was employed. The expression of the chimeric Pfs16-GFP allows for the analysis of parasite cultures by fluorescence activated cell sorting (FACS) enabling the accurate identification of gametocytes well before they are morphologically distinguishable from asexual stage parasites [17]. Initially, to quantify the effect of this compound on gametocytogenesis the time points at which these compounds had the greatest effect on gametocyte production was determined.

## Methods

### Parasites

Transgenic parasites (3D716-GFP) were maintained in culture and synchronized using 5% D-sorbitol as previously described [17]. Wild type 3D7 parasites were used for the MAS-Dansyl experiments.

### Gametocytogenesis assay

Cultures of ring stage transgenic parasites at 1% parasitaemia and 5% haematocrit (time 0 hrs) were dispensed in 250 μl aliquots in a 96-well plate in standard culture media, and exposed to culture media supplemented with either CT (Sigma) (10 μg/ml) or the peptide MAS 7 (Ile-Asn-Leu-Lys-Ala-Leu-Ala-Ala-Leu-Ala-Lys-Ala-Leu-Leu-NH2) (10 μM) for 48 hours. Parasites were also grown in the presence of 10 μM MAS 17 (Ile-Asn-Leu-Lys-Ala-Lys-Ala-Ala-Leu-Ala-Lys-Lys-Leu-Leu-NH2) which is an inactive derivative of MAS 7. Each culture was grown for a further 48 hours. Untreated control parasites were grown in standard culture media for the entire 96-hour assay period. Samples (50 μl) were taken from each well of the test and control groups at 0 and 96 hours and stained with 15 μg/ml ethidium bromide (EtBr) for 10 minutes at 37°C. Cells were washed three times with PBS pH 7.4, centrifuged for 1 minute at 180 × g and transferred to 5 ml FACS tubes for analysis. The number of GFP/EtBr positive (gametocytes) and EtBr only positive parasites (asexuals) were counted from a total of 5,000 EtBr positive cells. The number of gametocytes present at 0 hours was subtracted from the number of gametocytes present at 96 hours to obtain the total number of gametocytes produced during the 96-hour assay period.

### Time course studies

In order to define the time of sensitivity to MAS 7 or CT induced stimulation of gametocytogenesis within the 48-hour asexual cycle prior to development of gametocytes, cultures containing ring stage parasites underwent two rounds of sorbitol synchronization (4 hours apart) and were treated with either 10 μg/ml cholera toxin or 10 μM MAS 7 for 6 hour windows across the 48-hours lifecycle. At the end of each 6-hour incubation period the cells were washed 3 × in standard culture medium and cultured to the end of the 96-hour assay period. Samples (50 μl) were taken from each well of the test and control groups at 0 and 96 hours stained with 15 μg/ml EtBr for 10 minutes at 37°C and processed as described above to determine the number of parasites undergoing gametocytogenesis during the toxin treatment period.

### Furosemide treatment

Cultures were grown to trophozoite stage and treated for 12 hours with CT (10 μg/ml), MAS 7 (10 μM), or MAS 17 (10 μM) alone or in combination with 100 μM furosemide. After this treatment parasites were maintained in culture for a further 24 hours before being prepared for FACS analysis by washing in PBS. FACS analysis was performed at time 0 to quantify the initial gametocyte count and then again at 48 hours to provide the final gametocyte count.

### cAMP enzyme immunoassay

cAMP assays were conducted using a cAMP Biotrak enzyme immunoassay (EIA) system (GE Healthcare). Briefly, 6 ml cultures of trophozoite stage 3D716-GFP parasites at 5% parasitaemia were treated with CT (10 μg/ml), MAS 7 or MAS 17 (10 μM) for 30 minutes. Following treatment samples were analysed according the manufacturer’s instructions. Untreated cultures of infected (3D716-GFP) and uninfected red blood cells were included as controls.

### Immunofluorescence

MAS 7 and 17 peptides were synthezised and conjugated with the fluorescence tag Dansyl. Synchronized ring stage 3D7 parasites were incubated overnight at 37°C in the presence of 10 μM MAS 7/17-Dansyl peptides with and without the lipid dye BODIPY-TR-ceramide (1 μM). The cells were then prepared for microscopy as previously described [18]. Briefly, labeled cells were immobilized on slides with Concanavalin A (0.5 μg/ml) and fixed for 20 minutes in 2% Paraformaldehyde, 0.008% Glutaraldehyde. After fixation the slides were washed in 1 × PBS and the sample mounted for microscopy. Short-term uptake experiments were performed on 30–34 hour trophozoite stage parasites. MAS 7/17 was added to complete culture media and incubated for 1 or 2 hours at 37°C. Cells were washed in RPMI-HEPES and live infected RBCs were mounted and viewed directly without fixation. Microscopy was performed on a DV Elite wide filed deconvolution microscopy system (Applied Precision). Images were prepared using NIH ImageJ version 1.42.

## Results

The effect of the peptide MAS 7 on commitment to gametocytogenesis in the transgenic *P. falciparum* line 3D716-GFP was determined and compared to the effect of cholera toxin (CT). The 96-hour assay duration allows for the commitment rate of a single generation of parasites to be assessed as commitment to gametocytogenesis has been shown to occur one cycle prior to the detection of gametocytes [5,19]. The number of gametocytes present at 0 hours was subtracted from the number of gametocytes present at 96 hours to obtain the total number of gametocytes produced during the 96-hour assay period.

**Figure 1:**
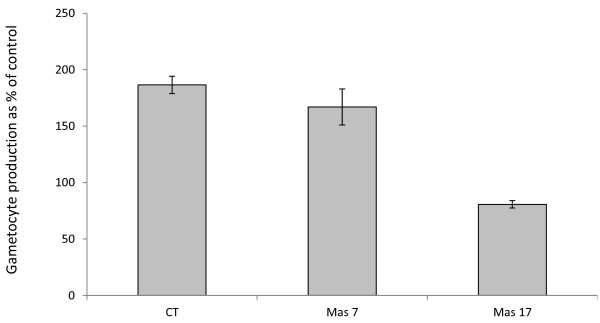
**The effect of cholera toxin (CT), MAS 7, or MAS 17 on commitment to gametocytogenesis was determined using the transgenic *Plasmodium falciparum *line 3D716-GFP.** Gametocyte production is expressed as a percentage relative to untreated control cultures. (N = 9).

Cells treated with CT showed a 186% increase in commitment (mean number of gametocytes = 83 ± 7.7) compared with non-treated cells (mean number of gametocytes = 44.5 ± 6.4) (Figure 1) which is slightly lower than rates demonstrated by Dyer and Day [5] but is still comparable with their findings. In mammalian systems CT is an activator of the Gαs which activates adenylyl cyclase leading to signalling through the secondary messenger cAMP. If acting canonically it catalyzes the ADP-ribosylation of the α_s_ class of heterotrimeric G protein α subunits and it has been reported to detect a number of G_α_ subunits in *P. falciparum-*infected red cells [13].

In mammalian systems the Gαs and Gαi/o signalling pathways generally act in opposition to each other with the Gαs pathway activating and the Gαi/o pathway inhibiting adenylyl cyclase signalling. As it was demonstrated that CT does stimulate gametocyte production, if it were acting canonically through the Gαs pathway, MAS 7 would be expected to either down-regulate or have no demonstrable effect on gametocytogenesis via inhibition of adenylyl cyclase through the Gαi/o pathway. In fact it was found that cells treated with MAS 7 showed a 167% increase in commitment to gametocyte production (mean number of gametocytes = 189 ± 16) compared with untreated cells (mean number of gametocytes = 113 ± 13) (Figure 1).

To ascertain if the stimulatory effect seen with MAS 7 was due to a direct effect on the signalling pathway or if the observed effect was due to induction of a general stress response by the peptide, parasites were treated with MAS 17, which is an inactive derivative of MAS 7 created by altering two amino acids in the sequence of MAS 7 (L6K), (A17K). Parasites treated with MAS 17 did not show increased commitment to gametocytogenesis (mean number of gametocytes = 24 ± 3.3) when compared to untreated controls (mean number of gametocytes = 29 ± 3.7) (Figure 1) indicating that the effects seen with the active MAS 7 were not due to non-specific toxicity of the peptide. At 10 μg/ml CT had no significant effect on asexual replication in accordance with the findings of Dyer and Day [5]. However they did find that CT inhibited both asexual replication and gametocytogenesis at higher concentrations (50 μg/ml). Treatment with 10 μM MAS 7 and MAS 17 similarly had no effect on asexual replication with the parasitaemia increasing from 1% at the start of the assay period to a mean value of 7% at the end of the assay period.

**Figure 2:**
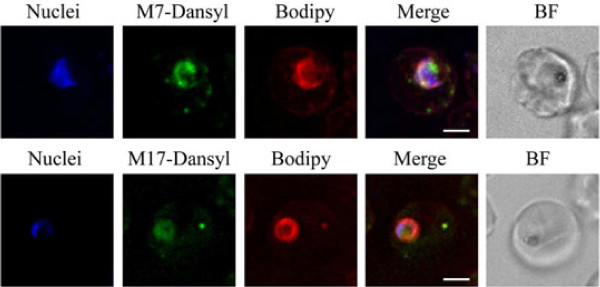
**Location of Dansyl-MAS 7 and MAS 17 peptides, within the parasite infected erythrocyte.** Parasite nuclei are shown in blue, Dansyl fluorescence in green and BODIPY-TR-Ceramide in red. A merge and bright field (BF) image are shown. Scale bar = 3 μm.

**Figure 3:**
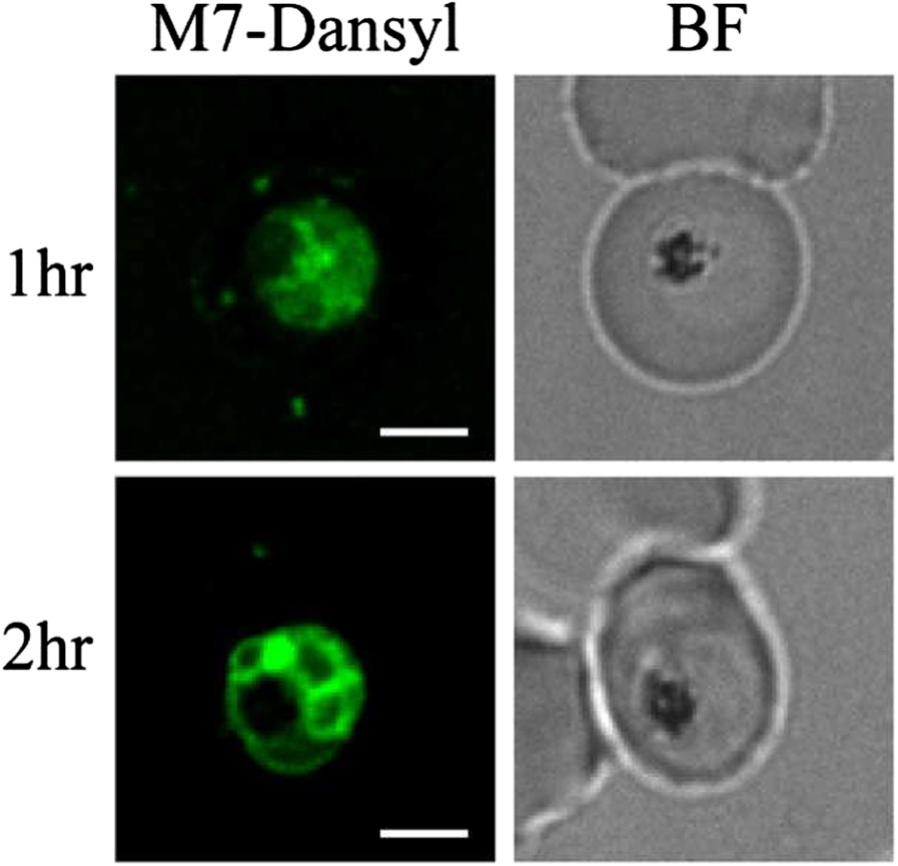
**Location of Dansyl-MAS 7 peptide, within the parasite infected erythrocyte following 1 hour (upper panel) and 2 hour (lower panel) incubation.** Scale bar = 3 μm.

In order to determine if the MAS 7 and MAS 17 peptides were acting at the RBC surface or if they were entering the RBC and the parasite, peptides conjugated to the fluorescence tag Dansyl were used. Co-labelling of cells with BODIPY-TR Ceramide showed that both the Dansyl-MAS 7 and MAS 17 peptides enter the infected erythrocyte where they appear as puncta within the RBC cytoplasm but with the majority of the peptide associating with the parasite, however the amount of MAS 17 appears to be less (Figure 2). The majority of parasites within the culture showed similar labelling patterns with no restriction of fluorescence to a sub-population of parasites. Labelling of the parasite cytoplasm was observed in trophozoite and schizont stage parasites consistent with findings showing maximal stimulation during these stages of development. No significant labelling was observed in ring and early trophozoite stage parasites. Uptake of the fluorescently labelled MAS 7 and accumulation at the parasite occurs within 1 hour of incubation (Figure 3). Accumulation of MAS 17-Dansyl was not observed at the parasite after 1 or 2 hours of incubation, and might reflect a limitation in the efficiency that the inactive MAS derivative can be taken up and accumulated at the parasite.

Dyer and Day [5] have previously shown that merozoites appear to be the stage of parasite which is susceptible to CT induced stimulation of gametocytogenesis in culture, so in order to define the time of sensitivity to MAS 7 induced stimulation of gametocytogenesis asexual parasites were pulsed at 6-hour intervals throughout the 48-hour asexual cycle prior to development of gametocytes, to determine if both compounds displayed a similar induction profile.

**Figure 4:**
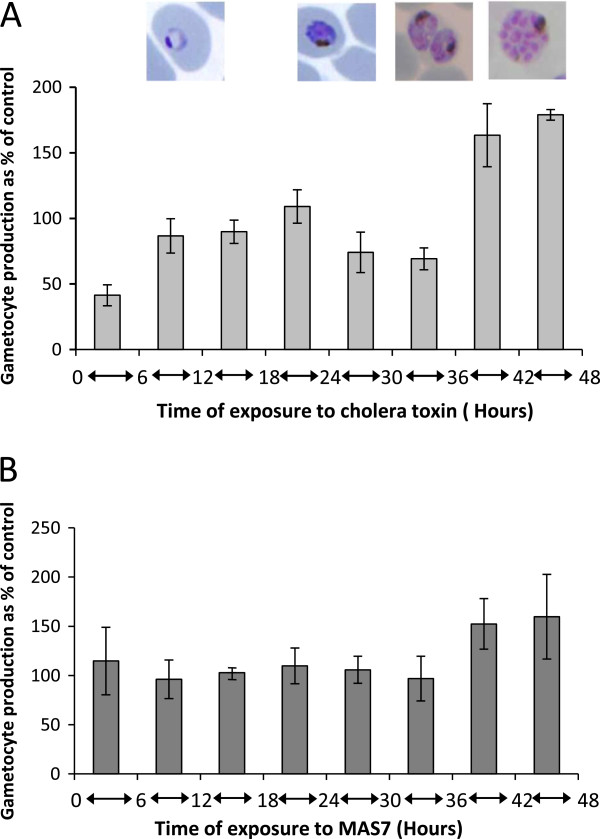
**Exposure of asexual parasites to cholera toxin and MAS 7 results in significant stimulation of commitment to sexual development.** The time of sensitivity to cholera toxin (**A**) and MAS 7 (**B**) induced stimulation of gametocytogenesis within the 48-hour asexual cycle prior to development of gametocytes. Gametocyte production is expressed as a percentage relative to untreated control cultures.

Exposure to both cholera toxin and MAS 7 during the later part of the parasite life cycle (36–48 hours) one asexual cycle before sexual differentiation resulted in a significant stimulation of commitment to sexual development with both compounds having similar induction profiles (Figure 4).

These observations confirm and extend the findings of previous studies. Dyer and Day [5] found that merozoites were most susceptible to cholera toxin induced stimulation of gametocyte production but also hypothesized that parasite stages other than free merozoites may be able to detect environmental stimuli using host cell signalling components and initiate commitment to gametocyte production. The observation that gametocyte production can be induced by stimulation as late as 36 hours into the lifecycle when the parasites would be late trophozoite/schizont stage support this hypothesis. Transcriptome data from *in vitro* cultures indicate that expression of one of the identified *P. falciparum* adenlyl cyclases (PlasmoDb identifier - PF3D7_0802600) is maximal at this stage of the parasites asexual lifecycle.

**Figure 5:**
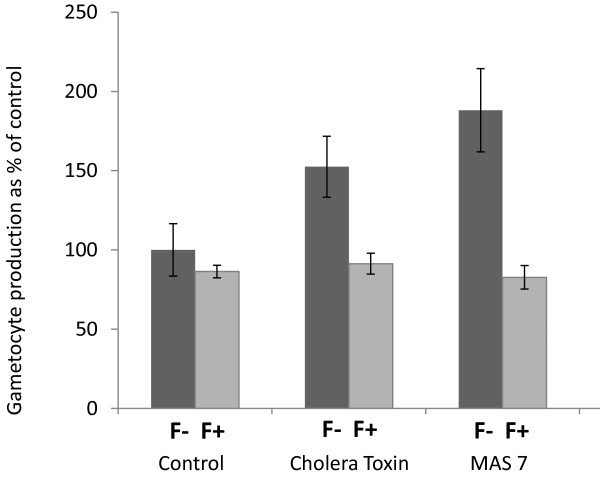
**The anion transport blocker furosemide, inhibits the gametocyte inducing activity of cholera toxin, and MAS 7 in the transgenic *Plasmodium falciparum *line 3D716-GFP.** Parasite cultures were grown to trophozoite stage and treated with cholera toxin or MAS 7 both with (F+) and without (F-) furosemide. Assays were performed in triplicate on two separate occasions and results of one representative experiment are shown.

A major anion transport pathway is initiated in the parasite-infected RBC in ring-infected erythrocytes with its activity peaking in the mature trophozoite and schizont stages [20]. This parasite induced pathway is known as the new permeability pathways (NPP) [21]. To investigate if the test compounds were entering the parasite via the NPP experiments with the anion transport blocker furosemide were undertaken. Cultures were grown to trophozoite stage (36 hours) and treated with CT, MAS 7, either alone or in combination with 100 μM Furosemide. Under these conditions furosemide led to a reduction in the number of detectable gametocytes (Figure 5).

Treatment with furosemide did not completely block gametocyte production when used in combination with CT and MAS 7. Furosemide-treated cultures produced similar numbers of gametocytes to the untreated (no CT or MAS 7) control. This may be due to the presence of parasites already committed to sexual development within the sample or alternatively, due to the toxicity of furosamide to asexual stage parasites. Cultures treated with furosemide showed a decrease in asexual parasitaemia during assay period (10.5% reduction in Furosemide/ CT treated cultures and 34% reduction for Furosemide/ MAS 7 treated cultures) and it is acknowledged that toxicity over the assay period must be taken into consideration when interpreting this data. Given these experimental difficulties it is not possible to state definitely if furosemide blocks commitment to gametocytogenesis or not, but this data does raise the possibility, albeit tentatively that these compounds are acting on the parasite as indicated by the uptake of Dansyl labelled MAS peptides into the parasite infected erythrocyte.

In order to further understand the changes induced by the addition of MAS 7 and CT to parasites and whether there were any downstream effects on the secondary messenger cAMP, the levels of cAMP were measured in *P. falciparum* parasites treated with CT, MAS 7 and its inactive form MAS 17. cAMP is known to be a key signalling molecule and has previously been implicated in *P. falciparum* sexual differentiation. Nevertheless, the experimental evidence is somewhat contradictory with researchers showing that cAMP can both stimulate [22] and inhibit [23] gametocyte production, there is some evidence for the involvement of cAMP-dependent pathway functioning in sexual differentiation in *Plasmodium*[4,24,25]. However it should be noted that the literature on cAMP in plasmodium is a very diverse body of results making comparison between studies difficult.

If CT functioned canonically in the parasite it should activate the Gαs pathway which in turn would activate adenylyl cyclase leading to signalling through increased levels of the secondary messenger cAMP. Treatment of parasites with MAS 7, which has been implicated as an inhibitor of the Gαs pathway, would have the effect of decreasing cAMP levels which is at variance with the observation that MAS 7 leads to an increase in gametocyte formation.

Treatment of trophozoite stage parasites with MAS 7 resulted in significantly higher cAMP levels (13.5 ± 4.5 pmol.) than in parasite cultures treated with CT (11.6 ± 2.5 fmol.) or the inactive peptide MAS 17 (7 ± 2.6 fmol.). These last two levels were similar to untreated controls, demonstrating that only treatment with MAS 7 leads to a significant increase in cAMP levels. The observation that CT treatment did not affect cAMP levels indicates that while addition of CT does increase gametocytogenesis, the method by which it induces increased commitment is not immediately obvious, except that is unlikely to be via heterotrimeric G proteins, either host or parasite derived, and may be due to a more general stress response upon addition of this toxin.

While the finding that MAS 7 induces an increase in cAMP level (instead of a reduction as in other cell systems) is quite preliminary, this observation is still noteworthy. However as MAS7 like CT acts via trimeric G proteins in other systems this result does not help elucidate how its action is related to the observed increase in gametocyte production. Nonetheless, the dramatic increase in cAMP levels seen with addition of MAS 7 indicates that this compound acts either directly or indirectly on the *P. falciparum* adenylyl cyclase. One further observation from the data is that while there was a 1000 fold increase in cAMP levels between the MAS 7 treated parasites and the untreated controls the number of gametocytes only increased approximately two fold. This may indicate that gametocytogenesis is relatively insensitive to fluctuations in cAMP levels or this particular parasite clone itself was relatively insensitive.

The steps leading to commitment to gametocytogenesis in *P. falciparum* are complex, experiments are technically challenging and the data often ambiguous making interpretation difficult. A further layer of complexity is added by the observation that human erythrocytes possess a signal transduction pathway which includes the heterotrimeric G proteins Gs and Gi, adenylyl cyclase and protein kinase A [26]. Incubation of erythrocytes with MAS7 stimulates both an increase in cAMP within the cell and ATP release [27,28]. The observed increase in cAMP accumulation following stimulation with MAS 7 was modest (22%) and no effect was seen on cAMP accumulation when erythrocytes were incubated with MAS 17. These observations are in line with the findings presented here.

G protein agonists and inhibitors have also been shown to have an effect on sporozoite motility and a potential role for G protein signalling in sporozoite motility has been documented. Albumin triggers motility in *Plasmodium* sporozoites [29], it interacts with the sporozoite surface initiating a signal transduction cascade including the elevation of cAMP which induces sporozoite motility [30]. Kebaier and Vanderberg [31] have shown that in the absence of albumin the addition of CT induced sporozoite motility while MAS 7 inhibits their motility. This inhibitory effect is not seen with MAS 17. While it is tempting to speculate that signalling in sporozoites and other blood stage parasites operates through the same pathways, this has not been definitively proven and a different mechanism may be operating.

## Conclusion

Gametocytogenesis by *P. falciparum* is essential for the parasite to complete its full lifecycle, and the data presented here adds one more piece to the puzzle of which pathways are likely to be involved in this process. Nonetheless, questions remain, if CT does not act by increasing cAMP levels where does it interact within the signalling pathway? CT also has been shown to increase the influx of Ca^2++^[32], which may suggest a possible mechanism. A precedent for this is provided by previous work using conditioned media, which suggested that a small molecule was responsible for the increased levels of gametocytogenesis seen in co-culture experiments [3]. The significant increase in cAMP seen with the addition of MAS 7 also indicate that the signalling mechanisms in *P. falciparum* are non-canonical and this may make the elucidation of the effector molecules that interact with the signalling pathway difficult to ascertain.

## Abbreviations

GFP: Green fluorescence protein; FACS: Fluorescence activated cell sorting; CT: Cholera toxin.

## Competing interests

The authors declare that they have no competing interests.

## Authors’ contributions

CLP, MWAD, DLG and KRT conceived and designed the study. CLP and MWAD undertook the experimental procedures. All authors participated in data analysis and interpretation. KRT drafted the final manuscript, which was read and approved by all authors.
